# Dentin contamination during repair procedures: A threat to universal adhesives?

**DOI:** 10.1002/cre2.551

**Published:** 2022-03-08

**Authors:** Anne‐Katrin Lührs, Cosima Brachmann, Silke Jacker‐Guhr

**Affiliations:** ^1^ Department of Conservative Dentistry, Periodontology and Preventive Dentistry Hannover Medical School Hannover Germany; ^2^ Department of Prosthetic Dentistry and Biomedical Materials Research Hannover Medical School Hannover Germany

**Keywords:** adhesives, dentin, silanes

## Abstract

**Objective:**

This study examined the influence of surface contamination during repair procedures with hydrofluoric acid, silane, or ammonium polyfluoride on the bond strength of universal adhesives to dentin using different etching modes before and after thermocycling.

**Materials and Methods:**

Dentin surfaces of human molars were contaminated in different ways (silane, hydrofluoric acid, ammonium polyfluoride, and no pretreatment as control) followed by application of a universal adhesive (etch & rinse or self‐etch mode). After a composite build‐up was placed onto each tooth, sticks for the microtensile bond strength (MTBS) test were sectioned. Half of the sticks were tested after water storage for 24 h, the other half after thermocycling (15,000 cycles, 5/55°C). The MTBS data were analyzed statistically by using the Kolmogorov–Smirnov test, one‐way analysis of variance, and Tukey HSD test (*p* < 0.05). The fracture patterns of all specimens were evaluated and analyzed using a *χ* ^2^ test.

**Results:**

Dentin contamination with a multifunctional silane does not influence microtensile bond strength irrespective of aging. Contamination with hydrofluoric acid or an ammonium polyfluoride primer leads to a significantly lower bond strength after aging, irrespective of the etch mode.

**Conclusion:**

Dentin contamination with hydrofluoric acid or ammonium polyfluorides during repair procedures should be avoided, as they appear to decrease the bond strength of universal adhesives.

## 1 INTRODUCTION

Due to their high versatility, the use of universal adhesives for direct and indirect restorations is increasing (Perdigão & Swift, 2015). Indications include direct composite restorations, adhesive cementation of indirect restorations, and even surface treatments of metal restorations (Ikemura et al., 2012; Kanzow et al., 2019; Kim et al., 2015; Nagarkar et al., 2019). Universal adhesives contain various carboxylate‐ or phosphate‐based functional monomers, such as 10‐MDP, MCAP, GPDM, PENTA, 4‐MET, or PAC (Nagarkar et al., 2019). Also, universal adhesives are used more frequently to intraorally repair a wide variety of restorative materials (Demirel & Baltacioğlu, 2019; Kanzow et al., 2019). Currently, the proportion of repairs of existing restorations relative to all treatments performed in daily practice is rising (Kanzow et al., 2019). The advantages of repairs include the facts that they are minimally invasive, require less time in certain cases and allow clinicians to avoid the risk of damaging the pulp (Brunton et al., 2017). However, many repairs involve not only the restorative material but also to a variable extent the tooth structure. As the complexity of these clinical situations increases, it is often difficult to decide which approach to use, especially as there exist a variety of different recommendations for repair procedures in the literature, which are not consistent (Altinci et al., 2017). For instance, the surface pretreatment of ceramic for adhesive cementation and repair depends on its composition (Awad et al., 2017). For a successful cementation of a glass‐ceramic, for example, lithium disilicate, its surface has to be etched with hydrofluoric acid and coated with a silane (3‐methacryloxypropyltrimethoxysilane) (Tian et al., 2014). Applying only a universal adhesive after hydrofluoric acid etching, even if it contains a silane, cannot be recommended due to the instability of the silane component resulting in lower bond strengths, as compared to the conventional method, that is, hydrofluoric acid etching and silanization (Guimaraes et al., 2018; Maier et al., 2019; Yoshihara et al., 2016). When repairing restorations in clinical practice, adjacent enamel and dentin surfaces may be “contaminated” with agents used for restoration pretreatment. In the case of glass ceramics, this may apply to both hydrofluoric acid, which can be used intraorally for ceramic repairs in the form of buffered 9% hydrofluoric acid with strict safety precautions, and silanes (Hickel et al., 2013; Kanzow et al., 2019). Dentin contamination with a silane before the use of a universal adhesive does not seem to influence initial bond strength to dentin, neither in the self‐etch nor in the etch & rinse mode (Chen et al., 2017; Kanzow et al., 2020). However, enamel or dentin contamination with hydrofluoric acid should be avoided because it negatively affects the bond strength to the tooth structure (Loomans et al., 2010; Saracoglu et al., 2011).

The use of ammonium polyfluoride‐based primers is a new option for pretreating glass‐ceramic surfaces. Prior etching with hydrofluoric acid and subsequent application of silane is not necessary when using this pretreatment method, as the surfaces are etched by ammonium polyfluoride and silanized by trimethoxysilylpropyl methacrylate. These self‐etch “glass‐ceramic primers” provide bond strengths comparable to those achieved by conventional pretreatment with hydrofluoric acid and silanes, and therefore seem to be an interesting option for repairs of glass ceramic (Al‐Harthi et al., 2018; El‐Damanhoury & Gaintantzopoulou, 2018; Maier et al., 2019).

The null hypotheses of this investigation were set forth as follows:

(1) Surface contamination with hydrofluoric acid, silane or ammonium polyfluoride does not influence the bond strength of universal adhesives to dentin.
(2) The bond strength of universal adhesives to dentin does not differ between the self‐etch and etch & rinse mode, irrespective of the kind of contamination.
(3) The bond strength is not influenced by aging.

## 2 MATERIALS AND METHODS

**Figure 1:**
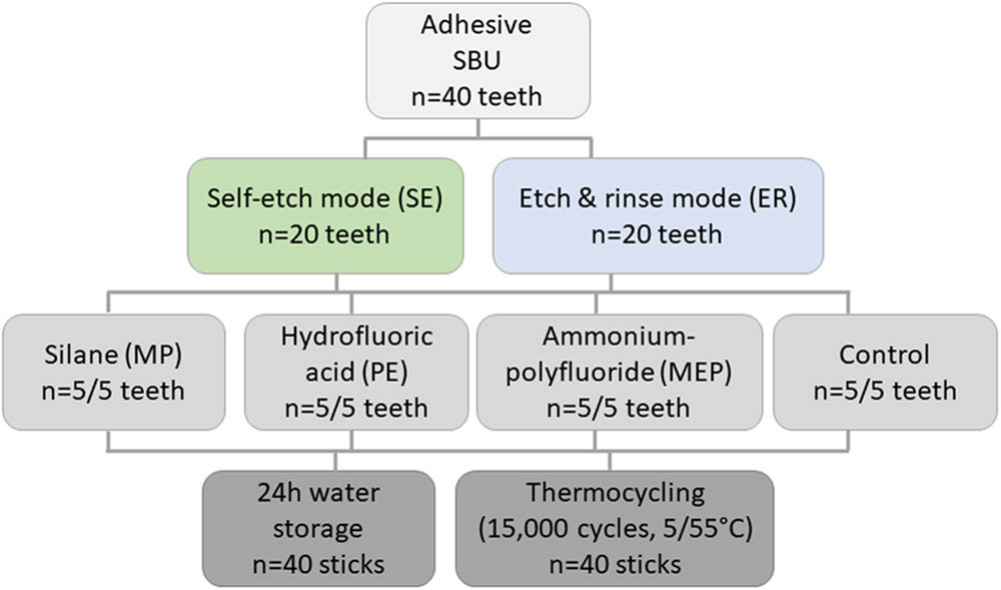
Test and control groups with their respective surface pretreatment (adhesive application [all groups] and contamination [test groups only]). Number of teeth per main group (*n*), resulting number of sticks per test group (*n*)

**Figure 2:**
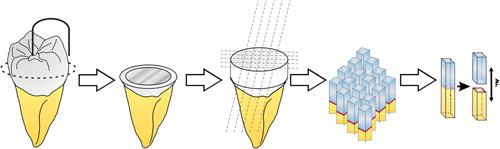
Illustration of the cutting procedure. Initially, a u‐shaped device was placed in the deepest part of the fissure in order to determine where the cut had to be placed. The upper part of the crown was cut was 1 mm below the deepest part of the fissure. After pretreatment of the dentin surface according to the respective treatment protocol and placing of the composite build‐up, five cuts in *x*‐ and *y*‐direction were made resulting in 16 sticks per tooth

For this study, 40 caries‐free human molars were used after obtaining the patients’ informed consent. The teeth were stored in 0.5% chloramine‐T solution at 8°C for no longer than 6 months before testing. The use of human teeth for bond strength testing has been approved by the Ethics Committee of Hannover Medical School (no. 2092‐2013). The teeth were randomly divided into eight main groups with each five teeth and kept moist throughout the testing period (Figure 1). The main groups were further subdivided into groups that were tested after 24 h water storage versus groups that were aged via thermocycling. All teeth were cleaned from debris and embedded in gypsum parallel to the tooth axis. A low‐speed saw (IsoMet Low‐Speed Saw; Buehler, Esslingen, Germany) was used under constant water cooling to separate the coronal part of the crown from the tooth at a right angle and expose the dentin. The cut was created 1 mm below the deepest part of the fissure. Therefore, a u‐shaped device was placed in the deepest part of the fissure in order to determine the height of the cut. The cutting process is illustrated in Figure 2. The dentin surface was then checked for any remaining enamel areas with dental loupes at ×4 magnification. When the surface still included enamel, slightly more tooth structure was removed until only dentin was exposed. The dentin surface was then roughened with moist abrasive paper (600‐grit, SiC Grinding Paper; Buehler, Esslingen, Germany) and thoroughly rinsed with water to create a clinically relevant smear layer.

Afterwards, the dentin surfaces were pretreated depending on the groups the teeth had been assigned to. In half of the test groups, the universal adhesive was used in the self‐etch mode, and in the other half in the etch & rinse mode. The universal adhesive was applied as described in Table 1. In the self‐etch mode, the adhesive was applied to the dentin, carefully rubbed onto the surface for 20 s, and gently air‐dried for approx. 5 s; in the etch & rinse mode, the dentin was etched with 35% phosphoric acid for 15 s, rinsed with water for 15 s and gently dried, before adhesive application. The surfaces were “contaminated” with silane, ammonium polyfluoride or buffered hydrofluoric acid in the test groups (Figure 1). In the self‐etch mode, the dentin was contaminated directly before adhesive application, and in the etch & rinse mode, between phosphoric acid etching and adhesive application. In the control groups (ER and SE), the dentin did not undergo any contamination. The universal adhesive was light‐cured with an LED unit (Bluephase; Ivoclar Vivadent, Schaan, Liechtenstein) for 10 s (output > 1000 mW/cm^2^). Before each polymerization cycle, the light output of the unit was checked with a radiometer (Bluephase Meter; Ivoclar Vivadent, Schaan, Liechtenstein). Then, a composite build‐up (Z100 MP Restorative, shade A3, 3M Oral Care; 3M Deutschland GmbH, Seefeld, Germany) was placed onto each tooth in three layers of 2 mm. Each layer was light‐cured from the occlusal aspect for 20 s (output > 1000 mW/cm^2^). Following the polymerization of the final layer, each of the four lateral surfaces were also light‐cured for 20 s, so that the total polymerization time of the composite buildup was 140 s. Afterward, the teeth were sectioned with a computerized high‐precision saw (IsoMet High‐Speed Pro; Buehler, Esslingen, Germany) in order to obtain sticks for the microtensile bond strength (µTBS) test. For each tooth, five cuts in *x*‐ and *y*‐direction were made resulting in 16 sticks per tooth (80 sticks per the main group). Half of the sticks (*n* = 8) obtained from each tooth were tested after storage in water at 37°C for 24h (*n* = 40 sticks), the other half after thermocycling (15,000 cycles, dwell time 30 s, changeover time 10 s, 5°C/55°C, *n* = 40 sticks). Specimens that fractured during sawing or thermocycling were included in the statistical analysis as “zero bonds.” All specimens were carefully measured with a digital caliper before testing, and the size of the bonded interface area was documented. For µTBS testing, the sticks were fixed to a universal testing machine (MTD‐500 plus; SD Mechatronik, Feldkirchen‐Westerham, Germany) using a cyanoacrylate glue (Roxolid Aktiv‐X; Hornbach AG, Bornheim, Germany) and loaded at a crosshead speed of 0.5 mm/min until fracture. Bond strength (MPa) was calculated by dividing the maximum force (N) measured and recorded for each specimen by the bonded interface area (mm^2^).

**Table 1:** Materials, manufacturers, compositions, and application techniques

Material	Manufacturer	Composition	Application	LOT No.
Scotchbond Universal (SBU)	3M, 3M Oral Care, 3M Deutschland GmbH, Seefeld, Germany	10‐MDP, HEMA, dimethacrylate, Vitrebond copolymer, filler, ethanol, water, initiators	SE: Apply Scotchbond Universal adhesive, rub in carefully for 20 s with a brush, and gently air‐dry for approx. 5 s until no movement of the liquid is visible any longer.	80912B

			ER: Etch dentin with 35% phosphoric acid for 15 s, rinse for 15 s and dry. For adhesive application and polymerization see SE mode.	
Porcelain Etch (PE)	Ultradent, Brunnthal, Germany	Hydrofluoric acid, 9%	Leave undisturbed for 60 s, thoroughly rinse with water and dry with air.	BC7PZ
Monobond Plus (MP)	Ivoclar Vivadent GmbH, Schaan, Liechtenstein	Methacrylate phosphoric acid ester, ethanol, silane	Apply Monobond Plus to dentin with a microbrush, leave undisturbed for 60 s (time needed to pretreat glass ceramics) and remove any excess with a strong stream of air.	Y09173
Monobond Etch & Prime (MEP)	Ivoclar Vivadent GmbH, Schaan, Liechtenstein	Tetrabutylammonium dihydrogentrifluoride, methacrylate phosphoric acid ester, butanol, bis(triethoxysilyl) ethane	Apply Monobond Etch & Prime to dentin with a microbrush, rub in for 20 s, leave undisturbed for another 40 s (times needed to pretreat glass ceramics), thoroughly rinse with water and dry with air for approx. 10 s.	X57010

Z100 MP Restorative–shade A3	3M, 3M Oral Care, 3M Deutschland GmbH, Seefeld, Germany	Silane treated ceramic, TEGDMA, BISGMA, 2‐benzotriazolyl‐4‐methyphenol	Apply three layers of 2 mm each.	NA15302

Abbreviations: 10‐MDP, 10‐Methacryloyloxydecyl dihydrogen phosphate; BISGMA, bisphenol A diglycidyl ether; ER, etch & rinse; HEMA, 2‐hydroxyethyl methacrylate; SE, self‐etch; TEGDMA, triethylene glycol dimethacrylate.

The fractures occurring in the μTBS test were assessed as described by Armstrong et al. (2017). Fractures located far (2 mm or more) from the interface in dentin, composite or cyanoacrylate were considered as missing values and not included in the statistical analysis.

Following the μTBS test, the fracture patterns of all specimens (cohesive in composite/dentin vs. adhesive at the interface vs. mixed) were evaluated with a light microscope at ×40 magnification. Then, the data were statistically analyzed using SPSS (Version 23.0; IBM Deutschland GmbH, Ehningen, Germany). First, the Kolmogorov–Smirnov test was used to check the data for normal distribution. Afterwards, a one‐way analysis of variance (ANOVA) and a Tukey HSD test was applied. The fracture patterns were analyzed using a *χ* ^2^ test.

To visualize any changes in the dentin surfaces, representative scanning electron microscope (SEM) images (Zeiss EVO 10 MA; Carl Zeiss Microscopy GmbH, Jena, Germany) were taken at ×1000 and ×3000 magnification before and after phosphoric acid etching and after contamination with hydrofluoric acid, silane, and the ammonium polyfluoride‐based primer.

## 3 RESULTS

### 3.1 Microtensile bond strength test

The one‐way ANOVA showed significant differences between the test and control groups (*p* < 0.001). The self‐etch (SE) and etch & rinse (ER) control groups did not significantly differ in initial bond strength (SE: 27.59 ± 9.92 MPa vs. ER: 25.52 ± 11.63 MPa; *p* = 1.000, Table 2). The values remained stable after thermocycling (TC) and were not significantly different (SE_TC: 25.10 ± 11.45 MPa vs. ER_TC: 26.55 ± 11.73 MPa; *p* < 1.000, Table 2).

**Table 2:** Mean microtensile bond strength in MPa and total number of sticks/zero bonds/samples excluded from statistics

	SE	ER	SE_TC	ER_TC
C	27.59 ± 9.92^aA^	25.52 ± 11.63^aA^	25.10 ± 11.45^aA^	26.55 ± 11.73^aA^
*n*/zero bonds/samples excluded from statistics	37/0/3	39/0/1	40/0/0	38/0/2
PE	29.74 ± 10.84^aA^	26.53 ± 15.05^aA^	10.65 ± 5.88^bB^	16.04 ± 6.22^bB^
*n*/zero bonds/samples excluded from statistics	36/0/4	36/0/4	37/0/3	34/0/6
MP	30.02 ± 12.98^aA^	27.90 ± 10.20^aA^	26.87 ± 13.42^aA^	24,59 ± 12.19^aA^
*n*/zero bonds/samples excluded from statistics	39/0/1	38/0/2	38/0/2	40/0/0
MEP	22.03 ± 10.41^aA^	29.60 ± 11.65^aA^	10.91 ± 5.97^bB^	15.82 ± 6.56^bB^
*n*/zero bonds/samples excluded from statistics	39/0/1	39/0/1	39/0/1	40/0/0

*Note*: Values with different lowercase letters in columns or different uppercase letters in rows are significantly different.

Abbreviations: ER, etch & rinse mode; MEP, Monobond Etch & Prime; MP, Monobond Plus; PE, Porcelain Etch; SE, self‐etch mode; TC, thermocycling.

Contamination of the dentin surfaces with buffered hydrofluoric acid (Porcelain Etch, PE), silane (Monobond Plus, MP), or ammonium polyfluoride (Monobond Etch & Prime, MEP) did not significantly influence the initial bond strength, irrespective of the adhesive application mode (SE vs. ER, Table 2). After artificial ageing by thermocycling, the bond strength in self‐etch and etch & rinse mode was significantly lower after contamination with hydrofluoric acid and ammonium polyfluoride when compared to the controls (SE_TC: 25.10 ± 11.45 MPa vs. PE_SE_TC 10.65 ± 5.88 MPa [*p* < 0.001] and MEP_SE_TC 10.91 ± 5.97 MPa [*p* < 0.001]; ER_TC: 26.55 ± 11.73 MPa vs. PE_ER_TC: 16.04 ± 6.22 MPa [*p* = 0.004] and MEP_ER_TC 15.82 ± 6.56 MPa [*p* < 0.001, Table 2]). Dentin contamination with silane did not significantly influence the bond strength in comparison to the controls, neither initially nor after thermocycling (Table 2).

### 3.2 Fracture analysis

The fracture analysis of all specimens tested by μTBS before and after thermocycling showed overall 64.0% adhesive, 5.7% cohesive, and 30.3% mixed fractures. After thermocycling, more adhesive fractures (52.2% before TC vs. 75.7% after TC) and fewer cohesive (7.6% before TC and 3.9% after TC) and mixed (40.2% before TC and 20.4% after TC) fractures were present (Figure 4). The differences in fracture patterns before and after aging were significant (*p* < 0.001).

The MEP_SE_TC test group showed the highest percentage of adhesive fractures (97.4%), and thermocycling led to an increase of 28.2%, as compared to the initial value of 69.2%. The difference between the MEP_SE and MEP_SE_TC groups was significant (*p* = 0.004). The MP_SE group showed the lowest percentage of adhesive fractures (28.2%). In this group, thermocycling also resulted in an increase in adhesive fractures of 35.0%–63.2%. These groups also had the highest percentages of cohesive fractures both before and after thermocycling (15.4% before TC and 18.4% after TC). The difference in fracture patterns between the MP_SE and MP_SE_TC groups was significant (*p* = 0.002).

### 3.3 SEM

The results of SEM are shown in Figures 5 and 6 (at ×1000/×3000 magnification). Dentin surfaces that had not been etched with phosphoric acid showed much more pronounced smear layers, irrespective of the kind of surface contamination (cf. Figures 5a–c and 6a–c). Neither silane nor hydrofluoric acid removed the smear layer at the microscopic level. Pretreatment with the ammonium polyfluoride primer, in contrast, seemed to superficially remove the smear layer, and dentinal tubule openings were visible, but sealed with precipitates (cf. Figure 6a,d). In all groups that included phosphoric acid etching (Figures 5e–h and 6e–h), the smear layer was removed, and open dentinal tubules were visible. Exposed surfaces contaminated with PE or MP looked much less homogeneous than uncontaminated surfaces (cf. Figures 6e compared to 6f,g). Surfaces contaminated with MEP resembled uncontaminated surfaces (cf. Figure 6e,h).

## 4 DISCUSSION

Due to their eligibility for all three etching modes (selective‐etch, etch & rinse, self‐etch), universal adhesives can be used for various purposes in clinical practice, including the repair of restorations. The aim of this in‐vitro study was to investigate the influence of dentin contamination on microtensile bond strength during a simulated repair procedure using universal adhesives.

**Figure 3:**
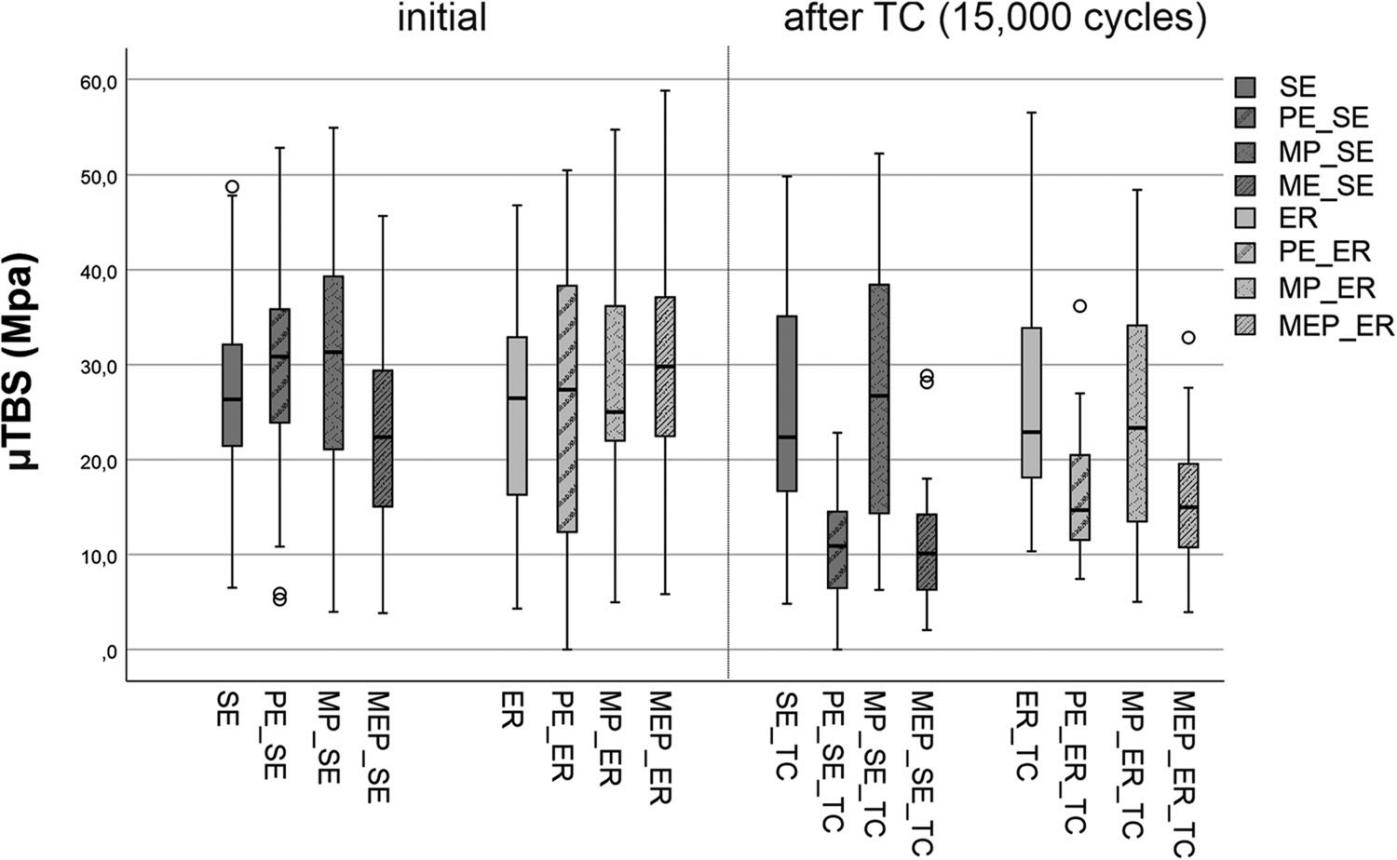
Boxplot demonstrating the microtensile bond strength (MPa) of the control and test groups after contamination (MEP, Monobond Etch & Prime; MP, Monobond Plus; PE, Porcelain Etch) to dentin initially and after thermocycling (TC) in either self‐etch (SE) or etch & rinse mode (ER). The median value is represented by the horizontal line within each box; the outliers are marked as circles

Regarding the methodology, the microtensile bond strength test was used to determine the group‐specific effects of different dentin pretreatments. Some groups in our study have a high standard deviation (cf. Table 2 and Figure 3). A high standard deviation might show a wider distribution of the measured values. This in turn could be associated with a higher uncertainty of the measured mean value. Nevertheless, a high number of specimens in each group (*n* = 40) was chosen in order to increase the validity of the method.

During adhesive procedures, dentin poses a greater challenge to adhesive bonding than enamel because of its composition (on average: 50% by vol. inorganic material, 30% by vol. collagen, and 20% by vol. water), and any negative influence resulting from dentin surface contamination may considerably affect bond strength (Perdigão, 2010). In this study, a universal adhesive containing 10‐MDP (10‐methacryloyloxydecyl dihydrogen phosphate) as a functional monomer was used. 10‐MDP is particularly suitable for restoration repairs because it is capable of bonding to oxide layers on the surfaces of zirconia ceramics and nonprecious alloys by means of hydrogen bonds (Nagaoka et al., 2017). Besides, this functional monomer can chemically bond to the tooth structure by forming a stable calcium salt with hydroxyapatite (Yoshida et al., 2004). For optimal bonding of 10‐MDP adhesive systems to dentin, manufacturers recommend carefully “rubbing” the adhesive into the surface to maximize monomer infiltration (Carrilho et al., 2019). Based on this recommendation (Moritake et al., 2019; Saito et al., 2020) the adhesive used in this study was also actively rubbed into the dentin surface with a brush for 20 s, gently air‐dried for 5 s, and light‐cured. This universal adhesive contains a silane, so it can theoretically bond to glass‐based ceramics (Matinlinna et al., 2018). However, it should be noted that silanes added to adhesive systems only have a positive influence on bond strength immediately after their addition, as they will be inactivated by a dehydration and condensation reaction after a very short time (Yao et al., 2018; Yoshihara et al., 2016). This is why a separate surface pretreatment with a silane is recommended when repairing glass ceramics, as any degradation processes at the interface can be prevented (Elsayed et al., 2017; Silva et al., 2020; Yao et al., 2018; Yoshihara et al., 2016). Concerning the intraoral repair of direct and indirect restorations, there have not been any consistent protocols to date, and the literature holds numerous and various approaches (Kanzow et al., 2019). During intraoral repair procedures, the tooth structure may be “contaminated” with a wide variety of repair agents. In a study conducted by Chen et al. (2017), no negative effect on initial shear bond strength to dentin was found, neither in the self‐etch nor in the etch & rinse mode, when using a universal adhesive after contamination with a silane (Chen et al., 2017). These findings are in accordance with the results of our investigation; the microtensile bond strength test did not show any significant influence of dentin contamination with silane on bond strength, neither initially nor after thermocycling. In addition, a study on this issue recently published by Kanzow et al. (2020) did not find any significant influence of bovine dentin contamination with silane before universal adhesive application in the self‐etch or etch & rinse mode. Using a universal adhesive in combination with a silane might be advantageous when glass‐ceramics need to be repaired and dentin is involved, as any contamination of the dentin surface with a silane will not negatively affect bond strengths (Table 2 and Figure 3). However, as the repair of glass‐ceramics not only requires silanization, but also the creation of surface roughness to increase the surface energy, silanization cannot be considered as an isolated factor, but only as a cofactor together with other pretreatment methods. Dentin contamination with ammonium polyfluoride (MEP) or hydrofluoric acid (PE) did not influence initial bond strength in our study. After thermocycling, however, the μTBS values after contamination with MEP or PE were significantly lower than those of the control groups. This was found irrespective of the adhesive application mode used (self‐etch vs. etch & rinse, cf. Table 2 and Figure 3). Therefore, the first null hypothesis has to be partially rejected because contamination with ammonium polyfluoride or hydrofluoric acid significantly influences bond strength. Since this effect occurred after thermocycling for these two agents, the third null hypothesis also has to be partially rejected. Similar results for dentin contamination with hydrofluoric acid after phosphoric acid etching were found by Saracoglu et al. (2011). The bond strength to contaminated dentin was significantly lower compared to the control group, but only 1000 thermocycles were performed, much fewer than in our study, and a classic two‐step etch & rinse adhesive was used instead of a universal adhesive. In contrast, another investigation showed that contamination with hydrofluoric acid does not significantly influence bond strength when a universal adhesive is used in the etch & rinse mode. In the self‐etch mode, however, bond strengths to contaminated dentin were significantly lower, which corresponds with the results of our study (Kanzow et al., 2020).

**Figure 4:**
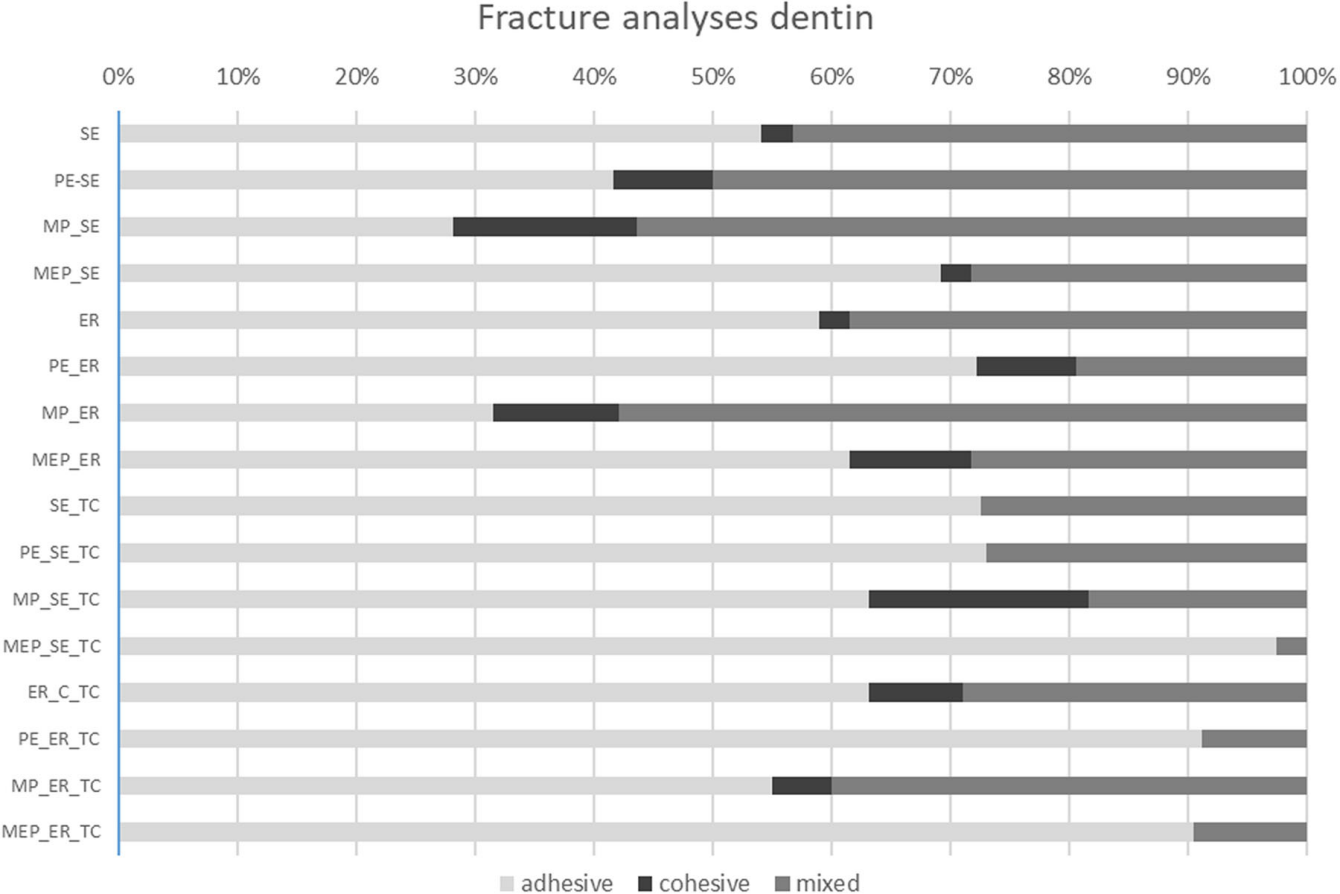
Fracture analysis of the control and test groups before and after thermocycling, a distinction between adhesive, cohesive and mixed fractured was made

The results of the fracture analyses support the findings of the μTBS‐testing. Groups with a high amount of adhesive failures were associated with lower bond strength values when compared to each other (cf. Figures 3 and 4). Especially after TC, where the dentin contamination with PE or MEP showed a significant influence on μTBS, the amount of adhesive failures was high (PE_SE vs. PE_SE_TC: 41.7% vs. 73.0% and PE_ER vs. PE_ER_TC: 72.2% vs. 91.2%; MEP_SE vs. MEP_SE_TC: 69.2% vs. 97.4% and MEP_ER vs. MEP_ER_TC: 61.5% vs. 90.5%). Therefore, a high amount of adhesive failure might be an indicator for the impact of the factors “contamination” as well as the “aging.”

**Figure 5:**
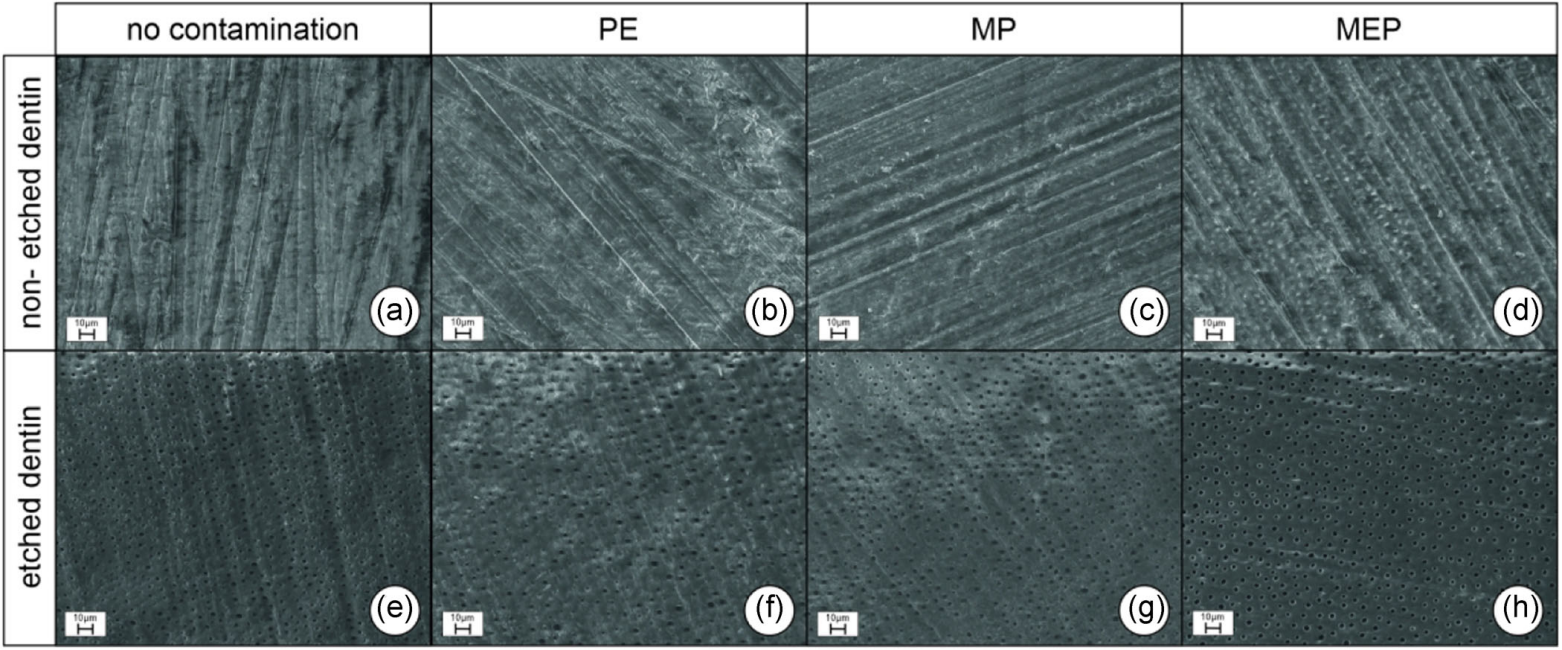
(a–h) Scanning electron microscope images of dentin surfaces at ×1000 magnification; (a–d): non‐etched, smear‐layer‐covered dentin without contamination and after contamination with hydrofluoric acid (PE), silane (MP) or ammonium polyfluoride (MEP); (e–h) analogous as described for (a–d), but dentin etched with 37% phosphoric acid, smear layer completely removed, dentinal tubules exposed. MEP, Monobond Etch & Prime; MP, Monobond Plus; PE, Porcelain Etch

Regarding the SEM analyses, the smear layer was not removed from the dentin surfaces by 60‐s “contamination” with hydrofluoric acid in our study (cf. Figures 5a vs. b and 6a vs. b), which is in consistence with the results published by Szep et al. (2000), who found an amorphous layer of the undefined precipitate. After etching dentin with phosphoric acid, in contrast, the smear layer was completely removed (cf. Figures 5e vs. f and 6e vs. f). The significantly lower bond strengths measured after thermocycling in comparison to the control groups cannot be attributed to the dentin structure visible with SEM. Nevertheless, EDX analyses showed that the application of hydrofluoric acid to nonetched dentin leads to an accumulation of fluoride ions in the surface (Szep et al., 2000). This accumulation might cause the fluoride ions to react with the residual calcium ions of the dentin surface to form CaF_2_ (Szep et al., 2000). As 10‐MDP also bonds to residual calcium ions, the reaction mechanism initiated by hydrofluoric acid application might inhibit this chemical bonding process. Loomans et al. (2010) also showed that bond strength to dentin significantly decreases after contamination with hydrofluoric acid and found certain changes in contaminated dentin via transmission electron microscopy. The changes included less demineralized dentin with a thinner hybrid layer. Also, the tubule openings were not widened by the hydrofluoric acid etching (Loomans et al., 2010). When dentin had been etched first with phosphoric acid and then with hydrofluoric acid, the hybrid layer was thinner, and there were mineral precipitates below the hybrid layer and areas with distinct nanoleakage (Loomans et al., 2010). These changes occurring at the microscopic or molecular levels might explain the lower bond strengths.

**Figure 6:**
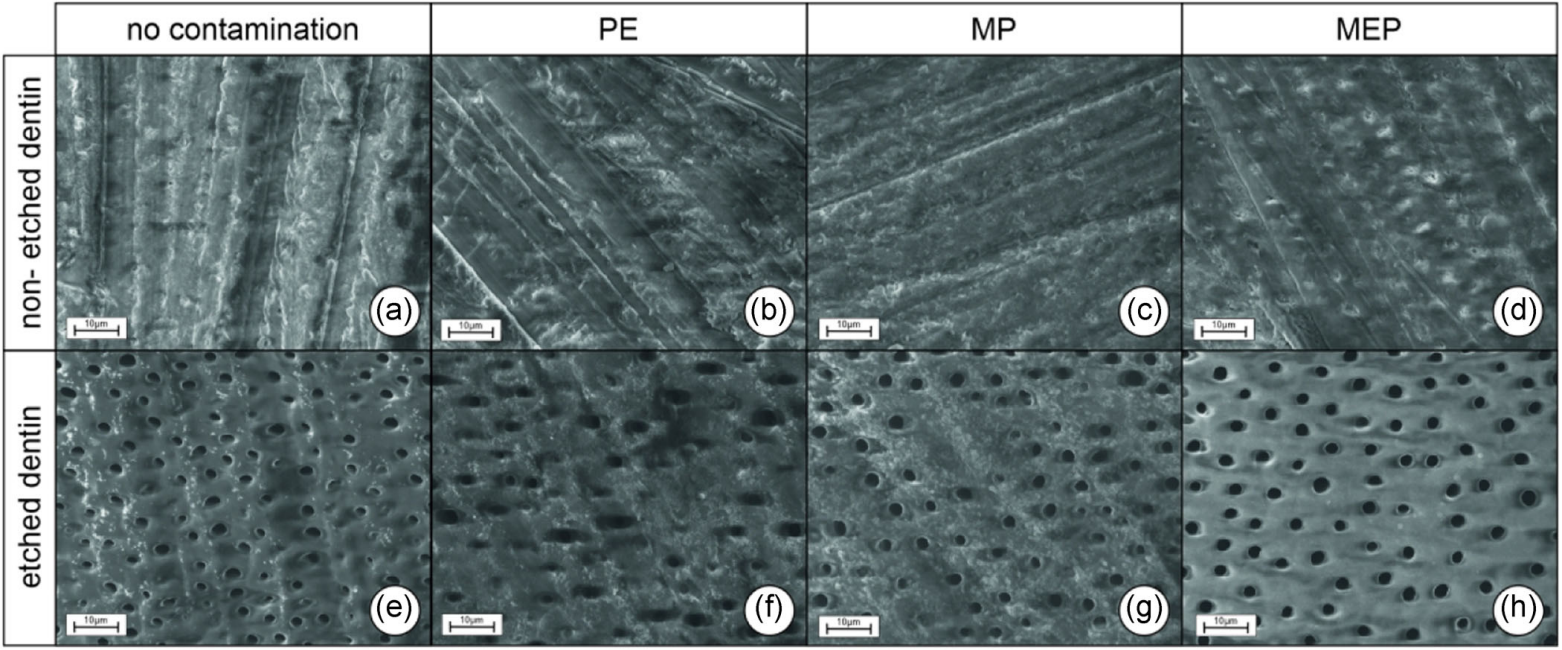
(a–h) Scanning electron microscope images of dentin surfaces at ×3000 magnification; (a–d) non‐etched, smear‐layer‐covered dentin without contamination and after contamination with hydrofluoric acid (PE), silane (MP) or ammonium polyfluoride (MEP); (e–h) analogous as described for (a–d), dentin etched with 37% phosphoric acid, smear layer completely removed, dentinal tubules exposed. MEP, Monobond Etch & Prime; MP, Monobond Plus; PE, Porcelain Etch

The use of ammonium polyfluoride primers for intraoral repairs of glass‐based ceramics could be an alternative to hydrofluoric acid. However, this kind of contamination also led to significantly lower bond strengths after thermocycling, as compared to the controls, irrespective of the adhesive application mode (self‐etch vs. etch & rinse, cf. Table 2 and Figure 3). In a shear test conducted by Kanzow et al. (2020) specimens that were contaminated with an ammonium polyfluoride primer and artificially aged did not differ from the control group when the etch & rinse mode was used, but for the self‐etch mode, a significant decrease in bond strength, comparable to our results, was found. Scanning electron microscopy showed that, without prior phosphoric acid etching, the smear layer was superficially removed by the use of the one‐component primer and the dentinal tubule openings were visible, but apparently sealed with precipitates (cf. Figure 6a vs. d). Similar results for this test group were described by Kanzow et al. (2020). In our study, the bond strength of the universal adhesive after contamination with the ammonium polyfluoride primer was significantly lower after artificial aging than that of the controls in both the self‐etch and the etch & rinse modes, so an interaction between the fluorides contained in the one‐component primer and the dentin surface at the molecular level, similar to the process described for hydrofluoric acid, may explain this decrease. However, as no other study in the literature describes such an interaction between ammonium polyfluoride primers and dentin, additional research will be necessary to clarify this phenomenon.

The second null hypothesis, stating that the bond strength of universal adhesives to dentin does not differ between the self‐etch and etch & rinse modes, irrespective of the kind of contamination, can be confirmed. When the dentin surfaces were contaminated with the same agent, there was no difference between the self‐etch and etch & rinse modes, neither initially nor after aging.

## 5 CONCLUSION

When repairing restorations intraorally, it is mandatory to differentiate between repairs limited to the restoration material and repairs involving the tooth structure. Dentin contamination with a multifunctional silane before the use of a universal adhesive does not influence bond strength irrespective of aging. Contamination with hydrofluoric acid or an ammonium polyfluoride primer leads to a significantly lower bond strength after aging. As the surface of glass‐based ceramic restorations has to be pretreated for establishing an adhesive bond, dentin contamination with substances involved in the repair procedure should be avoided.

## 6 CLINICAL SIGNIFICANCE

Repair procedures help to maintain the long‐term stability of dental restorations. During repair procedures, dentin contamination with hydrofluoric acid or ammonium polyfluoride primers should be avoided, as they impair the bond strength of universal adhesives after artificial aging.

## CONFLICTS OF INTEREST

The authors declare no conflicts of interest.

## AUTHOR CONTRIBUTIONS

*Study design & Methodology*: Anne‐Katrin Lührs. *Experiments*: Silke Jacker‐Guhr; Anne‐Katrin Lührs (in parts). *SEM‐Imaging*: Cosima Brachmann. *Statistical analyses*: Anne‐Katrin Lührs. *Creation of figures*: Anne‐Katrin Lührs. *Writing—original draft preparation*: Silke Jacker‐Guhr. Writing—review and editing: Anne‐Katrin Lührs, Silke Jacker‐Guhr.

## Data Availability

The data that support the findings of this study are available from the corresponding author, upon reasonable request.
